# Multifactor agitation: Several minor stresses severely compromise crop growth when combined

**DOI:** 10.1093/plphys/kiad608

**Published:** 2023-11-14

**Authors:** Kyle W Swentowsky

**Affiliations:** Assistant Features Editor, Plant Physiology, American Society of Plant Biologists; Cold Spring Harbor Laboratory, Cold Spring Harbor, NY 11724, USA

“The stresses imposed by climate change will limit agricultural production during the next century.” A version of this alarming but vague sentence can often be seen in news, media, and scientific journals, but precisely how climate change affects crops is not well understood due to the complex and variable ways climate change manifests. For simplicity, the impact of abiotic stress is usually evaluated using one stressor in isolation (e.g. how does drought stress affect growth?), but in a farmer's field, multiple stressful conditions often occur together, such as simultaneous heat and drought stress or flooding and high salinity during a tropical storm. Furthermore, the erratic nature of climate change events imposes unpredictable combinations of stressful conditions. How multiple stresses affect plant performance is called multifactorial stress combination (MFSC), and in this issue of *Plant Physiology*, Sinha et al. (2023) uncover how this phenomenon affects rice and maize.

It is intuitive that when multiple severe stresses are combined, a detrimental response is observed. However, what about the combination of multiple stresses that are individually too mild to elicit an obvious response? Ron Mittler's group initially began studying MFSC in Arabidopsis and found that when applied at low levels, six abiotic stresses (heat, salt, excess light, acidity, heavy metal, and oxidative) each had an insignificant impact on plant growth but severely limited plant productivity when multiple stressful factors were combined (Zandalinas et al. 2021a). The authors showed that MFSC elevated iron and reactive oxygen species (ROS) production, two types of molecules normally associated with stress responses. They also showed that ROS scavenging is required to mitigate the effects of MFSC. The study focused on the effects of MFSC in the model plant Arabidopsis, but it was not clear if combinatorial stress also affects crop species.

**Figure 1.**
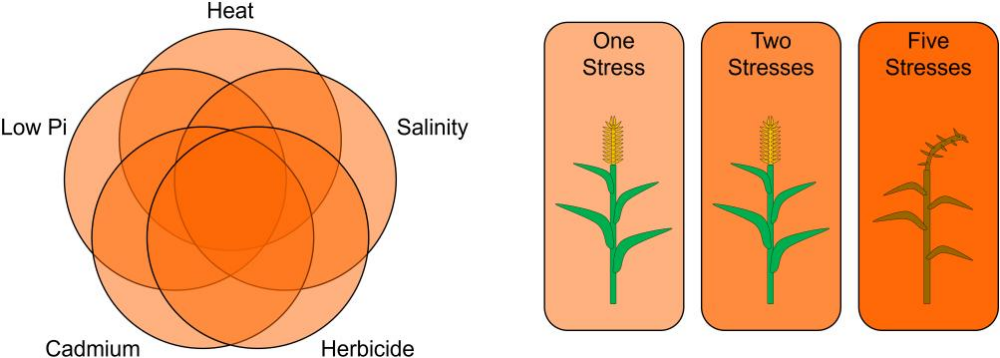
MFSC negatively affects crop performance when multiple low-level stresses are applied simultaneously. One (light orange) or two (medium orange) low-level stresses such as heat, salinity, herbicide, cadmium, and low phosphate (Pi) do not affect crop performance. When the same low-level stresses are applied together (dark orange), plant height, growth rate, biomass, and survivability are significantly compromised.

In the present study, Sinha et al. (2023) measured plant height, growth rate, and biomass in commercial varieties of rice and maize seedlings exposed to stress. When crops experienced low levels of five stresses individually (salinity, cadmium, the herbicide paraquat, heat, or phosphorus deficiency), growth was not noticeably affected. However, combinations of these stress conditions severely compromised plant growth by all metrics. Notably, while one or two stresses did not affect rice seedling survivability, four and five combined stresses reduced survivability to about 75% and 60%, respectively (Fig. 1). These results confirm that crop species are also susceptible to MFSC.

To understand how MFSC affects plant physiology so that we may develop strategies to mitigate these adverse effects, Sinha et al. (2023) next analyzed the elemental and protein compositions of rice seedlings exposed to multiple stresses. Rice seedlings accumulated significantly lower levels of nitrogen, phosphorus, potassium, and calcium when exposed to MFSC, but interestingly, magnesium and chlorophyll accumulation were not affected as they were in Arabidopsis (Zandalinas et al. 2021a). Proteomic analysis of rice seedlings revealed that particular low-level stress combinations (e.g. cadium/paraquat and cadmium/low phosphorus) led to greater changes in protein abundance than either treatment alone. Low-level heat stress alone had the greatest individual response and altered expression of over 1,500 proteins. A total of 332 differentially expressed proteins were common to all 4- and 5-stress combinations and were selected for further analysis. This set of proteins was enriched for gene ontology terms involved in different abiotic stress response activities, notably redox, ROS scavenging, and iron-sulfur metabolism. The findings are consistent with results from Arabidopsis (Zandalinas et al. 2021a) demonstrating that ROS and iron responses are common to MFSC in crop species. The authors speculate that engineering plants to mitigate the effects of ROS or iron homeostasis could be a potential mechanism to mitigate MFSC.

Next, the authors examined the natural variation of rice to uncover genetic diversity for resistance to MFSC. After screening 42 varieties of rice with diverse origins, three accessions showed moderate tolerance to MFSC. Interestingly, all three are the West African species, *Oryza glaberrima*, suggesting that genetic elements in this species enable robust growth under challenging climate change conditions.

Severe weather events, which have become more common, can impose stressful soil conditions, such as high levels of salinity or herbicides (IPCC 2021; Zandalinas et al. 2021b). The results presented by Sinha et al. (2023) demonstrate that crop species are reasonably adept at adapting to low levels of a single stress but become less resilient as the complexity of stress increases. The finding is a worrying prospect in the context of the unpredictable weather patterns imposed by climate change and highlights the need for future studies on MFSC and breeding more resilient crops.
